# Evaluation of the Influence of Machine Tools on the Accuracy of Indoor Positioning Systems

**DOI:** 10.3390/s222410015

**Published:** 2022-12-19

**Authors:** Till Neuber, Anna-Maria Schmitt, Bastian Engelmann, Jan Schmitt

**Affiliations:** Institute Digital Engineering (IDEE), University of Applied Sciences, Würzburg-Schweinfurt, Ignaz-Schön-Strasse 11, 97421 Schweinfurt, Germany

**Keywords:** indoor positioning systems, indoor gps, ultra-wideband, machine tools, accuracy, uncertainty, GUM

## Abstract

In recent years, the use of indoor localization techniques has increased significantly in a large number of areas, including industry and healthcare, primarily for monitoring and tracking reasons. From the field of radio frequency technologies, an ultra-wideband (UWB) system offers comparatively high accuracy and is therefore suitable for use cases with high precision requirements in position determination, for example for localizing an employee when interacting with a machine tool on the shopfloor. Indoor positioning systems with radio signals are influenced by environmental obstacles. Although the influence of building structures like walls and furniture was already analysed in the literature before, the influence of metal machine tools was not yet evaluated concerning the accuracy of the position determination. Accordingly, the research question for this article is defined: To what extent is the positioning accuracy of the UWB system influenced by a metal machine tool?The accuracy was measured in a test setup, which consists of a total of four scenarios in a production environment. For this purpose, the visual contact between the transmitter and the receiver modules, including the influence of further interfering factors of a commercially available indoor positioning system, was improved step by step from scenario 1 to 4. A laser tracker was used as the reference measuring device. The data was analysed based on the type A evaluation of standard uncertainty according to the guide to the expression of uncertainty in measurement (GUM). It was possible to show an improvement in standard deviation from 87.64cm±32.27cm to 6.07cm±2.24cm with confidence level 95% and thus provides conclusions about the setup of an indoor positioning system on the shopfloor.

## 1. Introduction: Indoor Positioning Systems

In recent years, the use of indoor localization techniques has increased significantly in different fields and applications, such as industry but also in healthcare, for monitoring and tracking reasons [1]. In the context of outdoor localization, the most well-known and widespread technology is the global navigation satellite system (GNSS). Due to the signal required from satellites, this technology is a good solution for outdoor use cases but is often unusable in indoor use cases due to the weakened signal inside of buildings, which results in inaccuracies in the determination of positions. A variety of different technologies exists for indoor localization in an industrial context. The range and accuracy, together with the cost of the system, are of central importance for an industrial use case [2].

### 1.1. Comparison of Radio Frequency Technologies

Indoor localization systems based on radio frequencies are the most widely used technology, mainly because of their comparatively low cost, high range, and ability to bypass obstacles in real use cases [2]. But even within the radio frequency technologies, there are major differences in terms of accuracy and range that need to be taken into account for the individual use case. Table 1 provides a general overview and comparison of the most important radio frequency technologies.

WLAN and Bluetooth technology offer a comparatively imprecise positioning at a high measurement range indoors. An ultra-wideband (UWB) system, on the other hand, can achieve significantly higher accuracy (an accuracy factor of ten more than WLAN) at an almost identical range. Ultrasound systems offer the highest precision in the field of radio frequency technologies (an accuracy factor of ten more than UWB), but their short range (max. 20 m) makes their use much more limited.

Industrial use cases have a high accuracy requirement for position determination (decimeter range) as well as a large production environment (>20 m). Systems that rely on UWB technology are therefore superior for the majority of industrial applications and therefore selected for the research work of this article.

### 1.2. Measuring Method and Characteristics of the Localino UWB System

**Figure 1:**
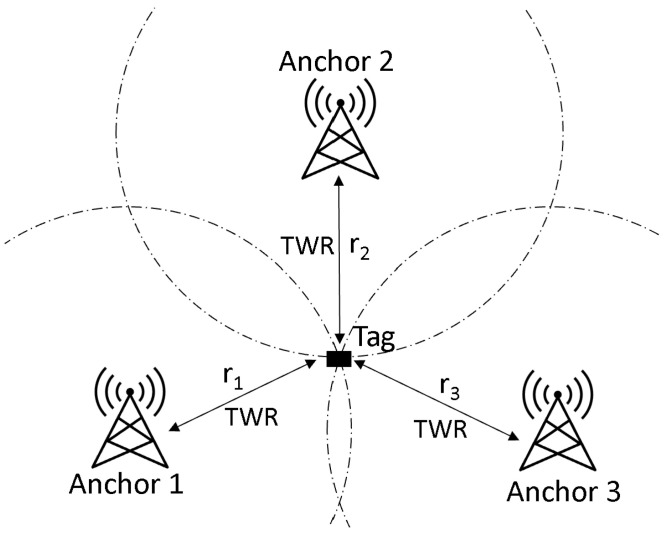
UWB measuring method [8].

In general, UWB technology is a short-range radio technology that uses relatively large frequency ranges (bandwidth >500 MHz) and thus enables low-interference tracking of people and objects indoors with low latencies [6]. The system consists of at least three transmitters (anchors), which are positioned at previously measured points, and a receiver (tag), which can be located in real time within the range of the transmitters. For localization in three-dimensional space, several steps are necessary, which can vary slightly depending on the specific measurement methods used by a manufacturer and are shown schematically in Figure 1. The UWB system Localino by the manufacturer Heuel & Löher GmbH & Co. KG (Lennestadt, Germany), which is used for the experiment, determines a distance measurement between the tag and the anchors (see Figure 1: r1, r2, r3) by using the two-way ranging (TWR) method. The distance between two devices is calculated by using the time a message signal travels from device A to device B and back to A [7]. As soon as at least three distances to different receivers are available, the tag position can be determined by using the trilateration method [8].

### 1.3. Related Work

In this section, a short overview of the recent research work in this field is given.

The received signal strength (RSS) is important for determining the coordinates of a tracked object. It can be affected by obstacles such as building architecture and walls [2], doors and windows [9], or furniture [10]. In [11] an improved wifi trilateration-based method for an indoor positioning system was proposed and evaluated inside of buildings. It was found that corridor wall obstacles can cause a positioning error in the x- and y-axis of 2.35 m and 1.25 m (with suggested compensation algorithms: x: 1.17 m and y: 0.6 m). In contrast to wifi-based indoor positioning, Alarifi et al. [12] point out that UWB technology offers advantages in terms of signal passing through obstacles. The low frequency of UWB pulses enables the signal to pass through walls and objects more effectively. However, this capability may be limited by power restrictions, which make it difficult to pass through walls. The effect of six-foot-thick concrete blocks and clay bricks on the electromagnetic radiation from UWB was evaluated by Zhu and Hong [13]. At a signal frequency of 10 GHz, a concrete block causes a two-way signal attenuation of about 30 dB and 6 db for clay bricks. The authors in [14] evaluate the signal attenuation of UWB on building materials as well as its interaction with a human body (concrete wall, 19 dB; dry wall, 10 dB; glass door: 9 dB; human body, 11 dB).

Location-based data can help in various shopfloor applications to improve the efficiency of processes. This data can be used to monitor and control orders by enhancing the information basis of a manufacturing execution system (MES), while in low automated production systems (e.g., the location of an order is entered manually into the system after finishing a production step). Location data can also help to interpret product and process information and allow visualization of disruptions and unpredictable events. [15]

Miller et al. [16] use location-based data to monitor the position and behavior of the machine operator, during the changeover process of a milling machine on the shopfloor. An industrial shopfloor environment can be characterized by industrial machines which are positioned in the production layout surrounded by corridors inside a building structure [17]. These environments also contain furniture, windows, brick or concrete walls, and massively built production machines on a shopfloor that consist of metal components. The relevance of metal obstacles for RFID-, UHF-RFID-, and UWB-based systems is pointed out in [18].

In [19], the tracking and monitoring of transporting trolleys within a industrial environment is evaluated. For Bluetooth low energy (BLE)-based technology the authors describe that the system availability decreased drastically in case of a metal object preventing clear line-of-sight (LOS) signal transmission. For UWB-based technology, their system was disturbed by reflected copies of the transmitted signal, which resulted in higher deviations for positioning measurements in case of non-line-of-sight (NLOS).

The described research work above shows that indoor location systems of different technologies are affected by obstacles in the measuring environment. For an industrial shopfloor environment, the focus of the research work concerning the influence of obstacles was put on walls, furniture, doors, and human bodies. The applicability of indoor positioning systems in a shopfloor environment is also influenced by machine tools being an important layout element of an industrial shopfloor. The influence of metal objects like industrial machine tools on the accuracy of positioning data has not been evaluated in detailed research work so far.

### 1.4. Research Question

As one of the most important properties of a UWB system, the accuracy is evaluated in a typical industrial use case on a metal machine tool. The research question is: To what extent is the positioning accuracy of the UWB system influenced by a metal machine tool?

To evaluate this question, a short introduction to the topic of measurement uncertainty and the presentation of the research methodology are given in Section 2. In Section 3, the experimental setup and approach are explained. This is followed by the discussion of the results in Section 4 with a final summary in Section 5, and a conclusion in Section 6.

## 2. Research Methodology

In addition to a general overview of measurement deviation types, a brief description of systematic influences on radio frequency technologies is given in this section. Subsequently, the research methodology for data acquisition and evaluation according to the guide to the expression of uncertainty in measurement (GUM) is explained.

### 2.1. General Measurement Uncertainty

**Figure 2:**
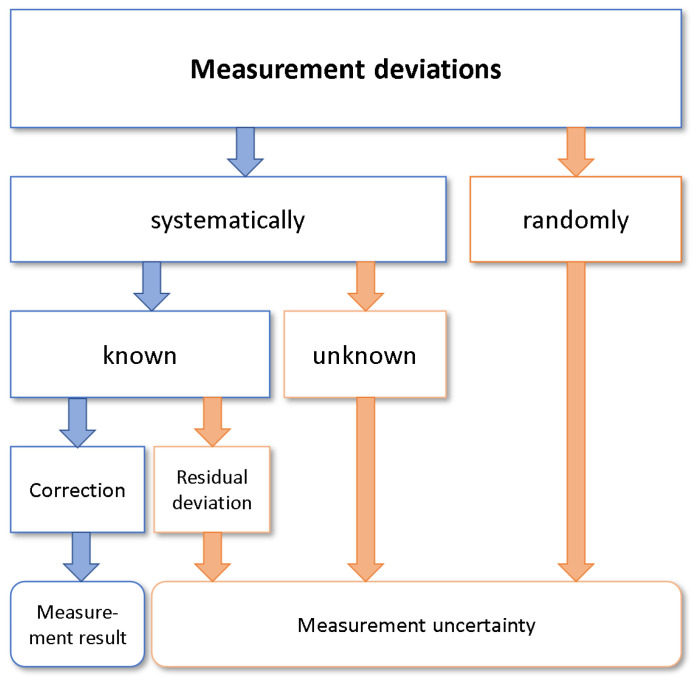
Overview of measurement deviations [21].

Generally speaking, the aim of any measurement is to determine the value of a specific measurand. However, the values measured by the measuring technique never exactly represent the “true” value, but only correspond to an approximation or estimation of this value [20]. The recorded value lies within a range around the true measurement result. This range is defined as measurement uncertainty and is composed of several types of measurement deviations [21]. Figure 2 shows these different types of measurement deviations schematically.

Measurement deviations can be divided into two basic categories: systematic and random measurement deviations. While random measurement deviations cannot be detected and corrected, i.e., they always contribute to the measurement uncertainty, some of the systematic measurement deviations can be detected and corrected. These known systematic measurement deviations result from a constant nonideal behavior of the signal processing of electronics and sensors. They can be detected by comparison measurements, also called calibration measurements. A simple repetition of the measurement is not sufficient. If necessary, the measurement can be improved with a small residual deviation by hardware and/or software correction methods [22]. Corrections of temperature influences or the removal of contamination can be regarded as examples [21].

### 2.2. Measurement Uncertainty and Influences on Indoor Positioning Systems

According to Liu et al. [23], the systematic measurement influences in radio frequency technologies can be divided into four categories: signal propagation, signal transmission, signal reception and signal processing (see Table 2). The signal propagation influences building structure, material, people, temperature and humidity in signal propagation were held constant during the experiment, indicated by the asterisk. The furniture placement is changed during the experiment. Main influences on the signal transmission are the anchor density, distribution, heights and models. The density and distribution factors are the nonconstant in this section. The density is determined by the number of anchors, which can be set to three or four anchors for the used system. In pretests, a setup with only three anchors showed such strong position deviations that it was decided to set the number of anchors constant to four. The local distribution of the anchors will be varied in four scenarios (see Section 3). Operators, receivers/tag, sampling, and number of samples are all constant factors of signal reception. For signal processing a fixed manufacturer specific filter is used in all experiments.

In a shopfloor environment the interfering objects are immovable. The focus of this research lies therefore on the layout of the transmitter modules.

### 2.3. Methodology of Data Collection

The aim of the following test setup is evaluating the systematic interference influences of machine tools on UWB systems by comparative measurements. For this purpose, a total of four different scenarios with a variation of the transmitter module positioning and the influence of interfering objects are carried out. A high-precision laser tracker measuring device of the Faro Vantage S6 type in the same coordinate system is used as a reference measuring device. Based on the recorded measuring points and the resulting deviations between the UWB system and the laser tracker measuring device, the data evaluation and knowledge derivation described in Section 2.4 is conducted.

### 2.4. Methodology of Data Evaluation According to GUM

The data evaluation is performed according to the type A evaluation of the standard measurement uncertainty from the guide to the expression of uncertainty in measurement (GUM). Here, the calculation of the arithmetic mean and the standard deviation of the measurement data is used [20]. For the arithmetic mean μ with *n* as the number of measured values $z_{i}$, the following Equation (1) is used:

(1) $$\mu =\frac{1}{n}\cdot \sum_{i=1}^{n}z_{i}.$$

For evaluation, the mean value is calculated based on the absolute deviations in each case for $z_{i}=\left|x_{i}^{L}-x_{i}^{R}\right|$, $z_{i}=\left|y_{i}^{L}-y_{i}^{R}\right|$ with $x^{L}$ as the measured value and $x^{R}$ as the reference value. The Euclidean distance $z_{i}={∥{\left(\begin{array}{cc} x_{i}^{R} & y_{i}^{R} \end{array}\right)}^{T}-{\left(\begin{array}{cc} x_{i}^{L} & y_{i}^{L} \end{array}\right)}^{T}∥}_{2}$ is used to evaluate the distance between the estimated position and the ground truth [24].

The standard deviation σ in Equation (2) is a measure for the deviation of the individual measured values $z_{i}$ from the arithmetic mean value μ and is calculated by the mean square deviation ([20], p. 43).

(2) $$\sigma =\sqrt{\frac{1}{n-1}\cdot \sum_{i=1}^{n}{\left(z_{i}-\mu \right)}^{2}}.$$

The standard deviation is calculated with the previously determined mean values for the x- and y-direction and the Euclidean distance for scenarios 1 to 4.

From the number *n* of individual measurements and the standard deviation σ of all absolute deviations of a scenario, the type A standard uncertainty $u_{A}$ can be calculated (see Equation (3)) [20] as

(3) $$u_{A}=\frac{\sigma }{\sqrt{n}}.$$

Finally, from the calculated mean value μ and the type A standard uncertainty $u_{A}$, the confidence interval (see Equation (4)) can be determined. The true value is located in the defined range of the confidence interval [25].

(4) $$Confidence\;interval:\mu \;\pm \;t\cdot u_{a}.$$

The parameter *t* depends on the selected confidence level and the number *n* of individual measurements and is taken from a predefined table [25]. For this research, the confidence level γ = 95% is used (see Section 3.5).

## 3. Setup and Approach

**Figure 3:**
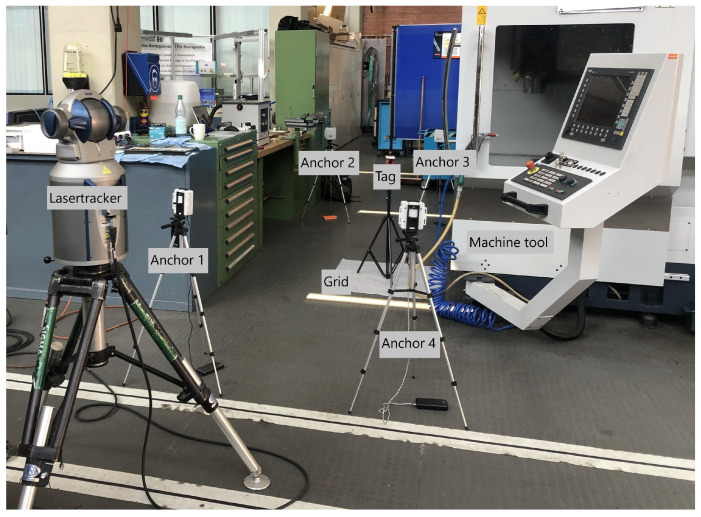
Setup with laser tracker.

The systematic influences of machine tools on the accuracy of UWB systems are measured and evaluated in the environment of a CNC machining center from the manufacturer Spinner in the laboratory of the University of Applied Sciences Würzburg–Schweinfurt (see Figure 3). A CNC machining center is a multiaxis machine that processes workpieces from batch size one by turning, milling and drilling processes and is part of the standard portfolio of many German small and medium enterprises [26]. The laboratory is located in an industrial machine hall in shed construction. The layout contains additional industrial machine tools and there are metal drawers and cupboards located next to the machine tool containing machine equipment (see Figure 3). In terms of equipment, the laboratory is thus comparable to a machine hall of a medium-sized manufacturing company.

As described in Section 1, the UWB system Localino, with four Anchor v4.0 modules from the manufacturer Heuel & Löher GmbH & Co. KG in TWR mode, is used [27]. For the test setup, the signal propagation was improved from scenario 1 to 4 by gradually increasing the active line of sight connections between the tag and the anchors, including the influence of other interfering objects on the indoor positioning system.

For flexible positioning within the scenarios, the anchor modules and the tag were set up by using five tripods, including suitable mounts and powered via four mobile power banks. The height of the positioning of both components was chosen to be as close as possible to the height of the employee’s trouser pocket and was therefore fixed at 90 cm. A Bosch–GLM 100-25 C PROFESSIONAL laser rangefinder with a measuring accuracy of ±1.5 mm was used to measure the anchor positions, which was necessary for the setup and operation of the UWB system [28].

To measure the deviation of the UWB system to the position on the grid, a different measuring instrument with higher accuracy is used. A laser tracker is one of the most suitable reference measuring devices for this use case due to its high accuracy, rapid deployment and widespread use in surveying [3]. In the test series, the high-precision reference data recording is therefore ensured with the help of the measurement service provider “sigma3D GmbH” and the laser tracker “Faro Vantage S6”, which can be seen on the left in Figure 3. The manufacturer FARO Europe GmbH specifies a measurement accuracy of 50 μm + 5 μm/m for the model with a maximum range of up to 80 m [29].

A list of all used devices in this article together with manufacturer and further device information can be found in Table A1 in the Appendix A of this article.

**Figure 4:**
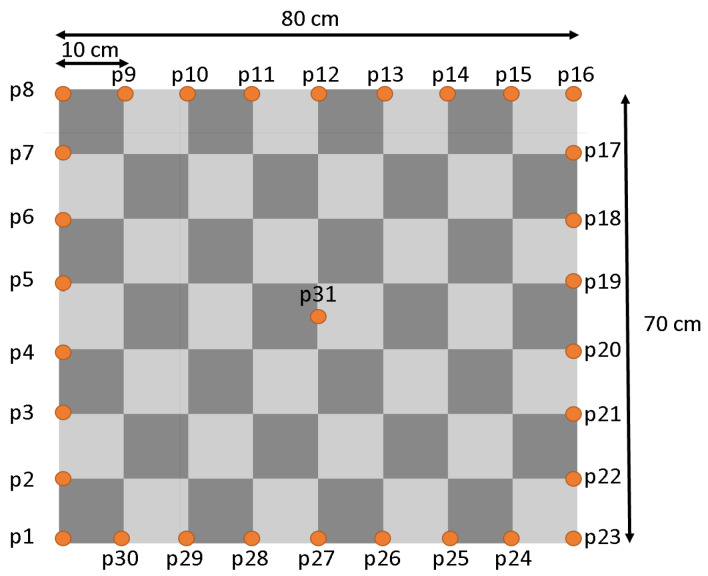
Grid measuring points.

In order to enable a repeatable and systematic measurement procedure in all scenarios, a similar procedure to the method described by Angelis et al. [30] is used for recording the measurement points. Here, the tag including the tripod is moved on previously defined points of a 1 m × 1 m grid. For the research work of this article, a commercially available pattern paper with a comparable size to [30] measuring 80 cm × 70 cm with a 10-cm grid was chosen. It is shown schematically in Figure 4.

The receiver (tag) was positioned on all points $p_{i}$ on the edge of the grid at 10-cm intervals including a central point results in a total of 31 measurement points recorded in each scenario.

### 3.1. Scenario 1

**Figure 5:**
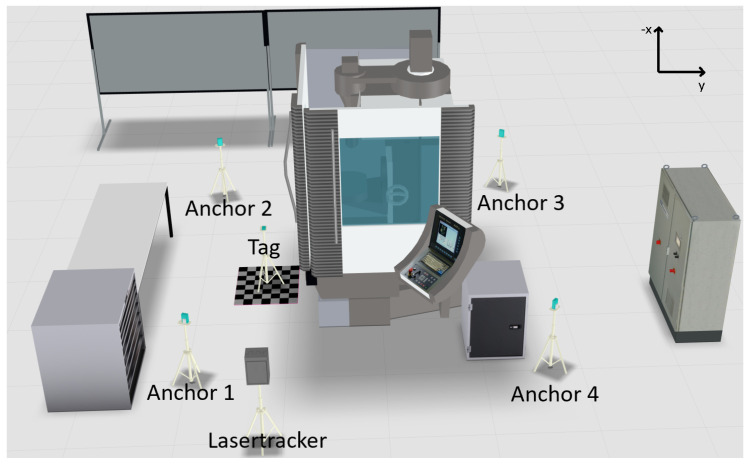
Visualization scenario 1: Two lines of sight.

In the first scenario, shown in Figure 5, the anchor modules are positioned in a rectangle around the machine, simulating a room with the modules in the corners. There are only two direct lines of sight to the transmitter and several objects are in the direct measurement range of the system that can potentially contribute to the measurement uncertainty. The laser tracker is at the same position in all scenarios and was referenced to the origin (anchor 1), $x_{min}$ (anchor 2) and $y_{max}$ (anchor 4) of the UWB system in each scenario in order to record the measurement data in the same coordinate system. The measuring point grid shown in Figure 3 stays at the same position for all measurements and is located with the row p8 to p16 at the door of the machine tool.

### 3.2. Scenario 2

**Figure 6:**
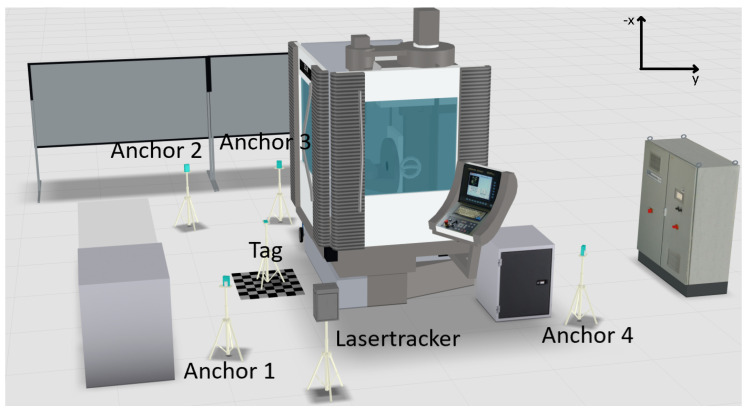
Visualization scenario 2: Three lines of sight.

In the second scenario, visualized in Figure 6, one of the anchor modules (anchor 3) is moved from a location without a line of sight to the tag to one with a direct line of sight to partially remove the impact of the machine tool on the localization. The positions of the other three modules remain identical to avoid further changes and potential influences on the system. In this scenario, other possible interfering objects in the measurement area exist.

### 3.3. Scenario 3

**Figure 7:**
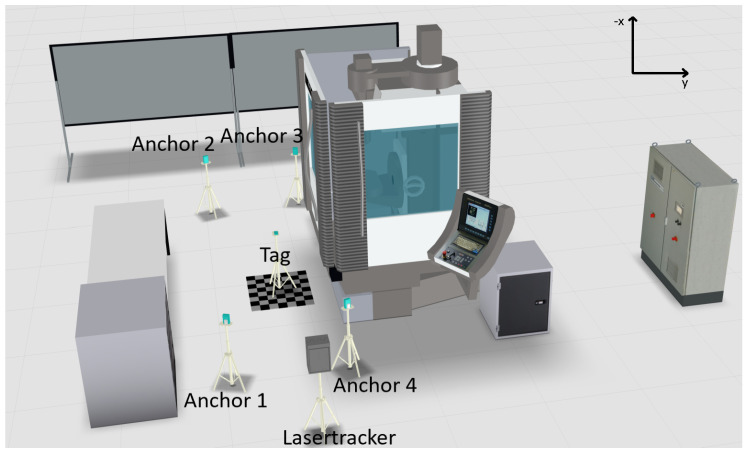
Visualization scenario 3: Four lines of sight.

In the third scenario, shown in Figure 7, the last anchor module (anchor 4) without visual contact is brought into line of sight of the receiver. The other anchor positions are not changed. Consequently, there are no other objects in the direct measuring range of the UWB system. Due to the short distances to the machine tool and other objects, further interference with the radio frequencies is possible (see Section 4).

### 3.4. Scenario 4

**Figure 8:**
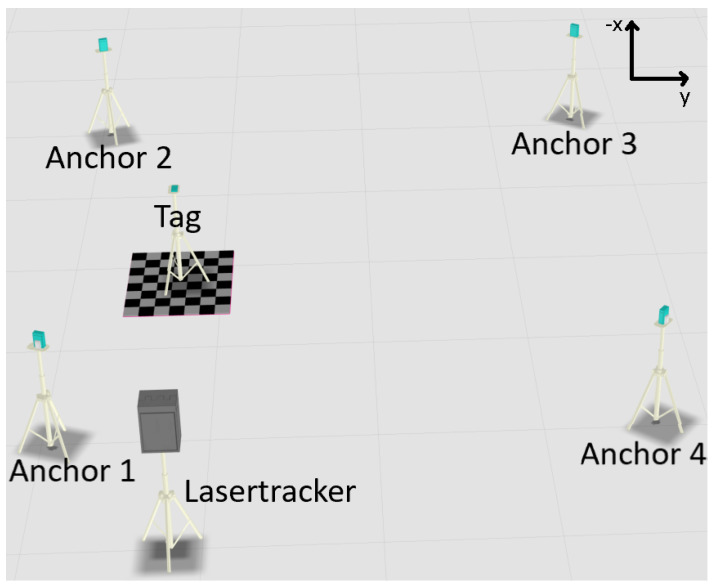
Visualization scenario 4: Four lines of sight in free environment.

Scenario 4, visualized in Figure 8, unlike the previous scenarios, does not take place directly at the machine tool, but in an environment without obstacles in the same industrial machine hall. In this scenario, the same system setup as in scenario 1 is used for the best possible comparability, but without further objects or interfering influences in and in the proximity of the system measurement area. This setup is chosen in order to generate the highest possible contrast to scenario 1.

### 3.5. Data Recording and Analysis

The anchor modules of the Localino system are connected wirelessly via an exclusive WLAN network, in which communication with the data-processing PC takes place. The Localino software enables real-time visualization of the position data, from which the recorded coordinates of the individual measuring points are exported into a comma-separated values (CSV) file format. The reference measurement data of the laser tracker are each saved by the measuring device in a CSV file format. For analysis, both CSV files are merged into a single file.

In the first step of the data analysis, a graphical visualization of the Localino measurement data for the four scenarios is performed. The next step consists of forming the absolute error of the x- and y-coordinates and the Euclidean distance between the measured Localino points and the laser tracker ground truth. This deviation is calculated for all 31 measurement points $p_{i}$ to visualize the difference between the measurements of the indoor positioning system and the reference measurements for the four scenarios. Based on these deviations, the arithmetic mean and the standard deviation are calculated for the x-coordinate, y-coordinate deviations and distances of a scenario, respectively, using the type A evaluation of the standard measurement uncertainty according to GUM, described in Section 2.4. As a result of fixing the height of the tag by using a tripod, the z-coordinate is not considered further in this experiment. In the third and last step, the confidence interval is calculated from the mean value and the standard deviation of all coordinate deviations of a scenario (see Equation (4)). The factor *t* is taken from a statistic table and equals t=2.05 for the number of measured values n=31 [25] (p. 295).

## 4. Results and Discussion

In this section, insights into the recorded measurement data gathered by the experiment described in Section 3 are presented. This is implemented by visualizing the indoor positioning system data in reference to the laser tracker data and by calculating the type A standard measurement uncertainty and confidence interval for all four scenarios (see Section 2.4). The complete measurement data is stored in a GitHub repository. The link can be found in the data availability statement at the end of the article. It was recorded one day by taking one measurement for each point.

The visualization performed in the first step serves as a general overview of the measurement data and is used to compare the scenarios with each other. For this purpose, the recorded x- and y-coordinates of the indoor positioning system (blue points; name “Lxx”) as well as those of the laser tracker (orange points; name “Rxx”) were displayed in a mutual coordinate system.

### 4.1. Scenario 1

**Figure 9:**
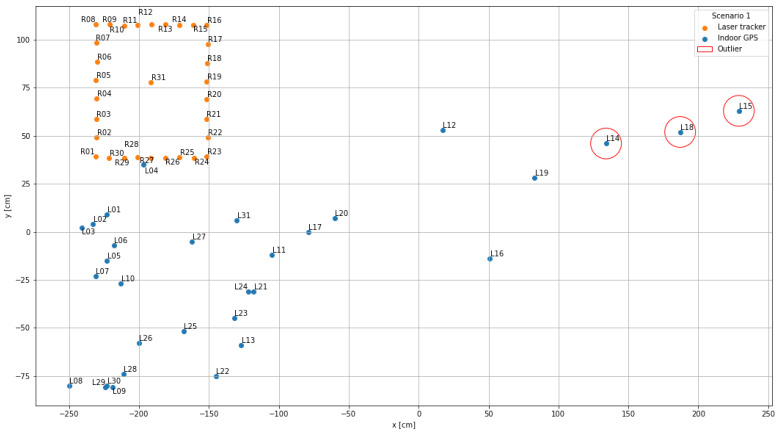
Data visualization scenario 1: Two lines of sight.

Figure 9 shows the first scenario with two lines of sight between the anchors and the tag. It displays a high deviation between the indoor positioning system measuring points and their corresponding reference points. This can be observed particularly for the points near the machine tool, especially L15 (deviation in x-direction: 390.4 cm), L18 (deviation in x-direction: 374.27 cm), and L14 (deviation in x-direction: 305.01 cm) as they are farthest away from the equivalent data points. The mean absolute error in x-direction is 75.02 cm and 91.11 cm in the y-direction. The mean error between the ground truth and the measured position is 141.18 cm. The corresponding standard deviations are 109.66 cm in the x-direction, 43.12 cm in the y-direction and 87.64 cm in total. The standard uncertainty calculated with Equation (3) is 15.74 cm resulting in the average deviation in this first scenario of 87.64cm±32.27cm with a probability of 95 % for the confidence interval.

### 4.2. Scenario 2

**Figure 10:**
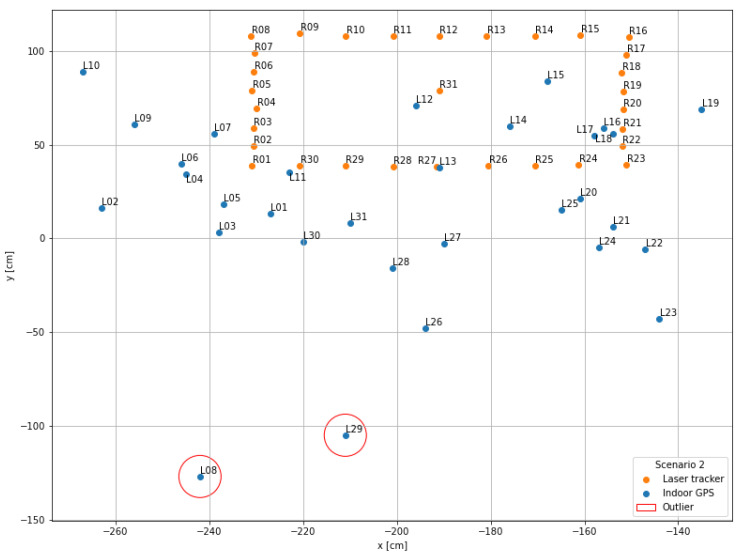
Data visualization scenario 2: Three lines of sight.

In comparison to scenario 1, the second scenario with three lines of sight, the measurements are closer to the reference points (see Figure 10). The measured points in the x-direction are closer to the reference points, the point with the highest x-coordinate is L19 with x=−135 cm compared to point L15 from scenario 1 with x=229 cm. In contrast, the y-coordinates decreased from y=−81 cm for points L09 and L29 of scenario 1 to y=−127 cm for point L08 of this scenario. On average, the calculated metrics are significantly smaller with the mean absolute error of 10.99 cm in the x-direction, 55.89 cm in the y-direction and 58.96 cm in total. The standard deviations for the x- and y-direction and in total are 11.97 cm, 41.48 cm and 40.30 cm, respectively. The confidence interval using the uncertainty $u_{A}=7.24$ cm is 40.30 cm ± 14.84 cm. In this scenario, there are only two major outliers: L08 (234.77 cm in y-direction; again near the machine tool) and L29 (143.64 cm in y-direction).

### 4.3. Scenario 3

**Figure 11:**
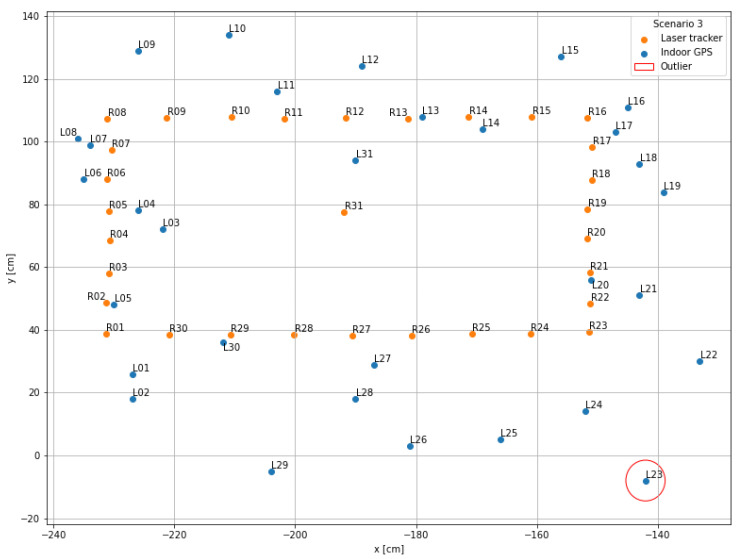
Data visualization scenario 3: Four lines of sight.

In the third scenario with four active line of sight connections, shown in Figure 11, the measurement point distribution of the indoor positioning system data could again be significantly improved in reference to the laser tracker data. Comparable outliers as in scenarios 1 and 2 no longer exist. The point L23 (y-deviation: 43.53 cm) has the largest deviation, and the indoor positioning system points result in a similar rectangular structure as the reference data. The mean errors are 5.37 cm in the x-direction, 15.99 cm in the y-direction, and 17.78 cm in total. The standard deviations are 3.97 cm, 12.82 cm, and 12.02 cm for the x- and y-direction and in total. The confidence interval in this scenario is 12.02 cm ± 4.43 cm with a probability of 95% for the confidence interval and 2.16 cm for the uncertainty $u_{A}$.

### 4.4. Scenario 4

**Figure 12:**
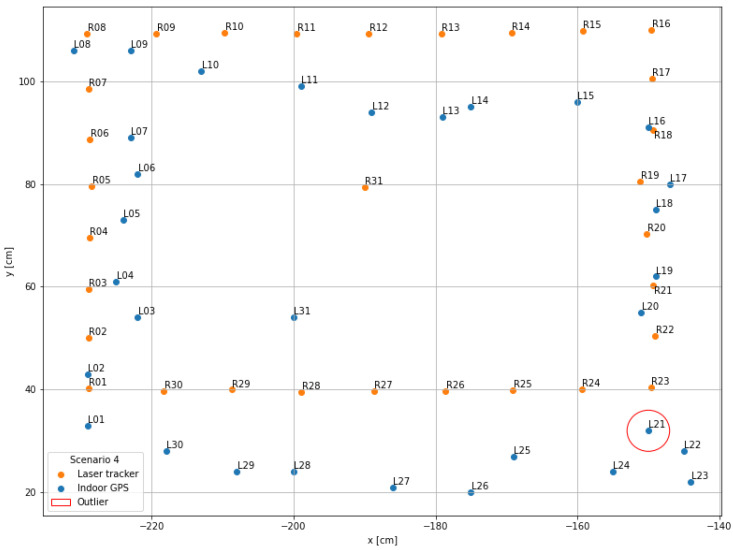
Data visualization scenario 4: Four lines of sight in a free environment.

The fourth scenario, shown in Figure 12, with four active lines of sight and no other objects or interfering influences in and immediately near the system measurement area, provides the best measurement point distribution of the indoor positioning system. There are no major deviations and the point L21 (y-deviation: 28.32 cm) is the furthest away from the reference point R21. The rectangular structure of the indoor positioning system measurement data is similar to the reference data. Along the y-direction, a minimal, systematic displacement of all indoor positioning system measurement points in relation to the laser tracker points can be observed. This observation is also evident in the mean absolute errors in the x-direction with 2.70 cm and 13.83 cm in the y-direction. In total, the mean error is 14.42 cm. The standard deviations are 2.58 cm in x-direction, 6.33 cm in y-direction, and 6.07 cm in total. The confidence interval in this scenario is 6.07 cm ± 2.24 cm with a probability of 95% for the confidence level and 1.09 cm for the uncertainty.

### 4.5. Comparison of the Scenarios

Table 3 shows a comparison of the four scenarios according to the methodology described in Section 2.4. The first three rows of the table contain the mean value of the deviations in the x- and y-direction and the total deviations of all scenarios. Based on these values, the standard deviations for the x- and y-direction and in total were determined in the third, fourth, and fifth rows. In the last two rows, the standard uncertainty and the confidence interval with a confidence level of 95 % were calculated based on the standard deviation and the number of measured values per scenario (n=31).

From scenario 1 (two lines of sight) to scenario 4 (four lines of sight in a free environment), the average deviation (confidence interval with a statistical certainty of 95 %) could be improved by a factor of ten from 87.64 cm ± 32.27 cm to 6.07 cm ± 2.24 cm. Adding one line of sight connection from scenario 1 with two lines of sight to scenario 2 to three lines of sight reduced the deviation by a factor of 2.2. The largest decrease is from scenario 2 (three lines of sight) to scenario 3 (four lines of sight) with a factor of 3.4. Although scenario 3 and 4 had the same number of line of sight connections, the mean deviation was also improved by almost 6 cm only due to the absence of interfering objects in the UWB system environment.

It is also noticeable that in almost every scenario the mean value and the standard deviation in the y-direction are significantly larger than in the x-direction. The exact cause has not yet been determined and may be a manufacturer specific behavior.

## 5. Summary of the Article

In Section 1, the relevance and the most important characteristics of indoor positioning system radio frequency technologies, including the research question, were presented. UWB systems are among the most suitable for industrial use cases with high precision requirements because of their low cost, high range, and accuracy in the decimeter range. The evaluated system Localino by the manufacturer Heuel & Löher GmbH & Co. KG was used to acquire measurement data using the TWR method.

In Section 2, after a general introduction of the types of measurement deviations including possible influencing factors, the most important systematically conditioned influences for radio frequency technologies were briefly discussed. In addition, the methodology of data acquisition and evaluation was described in this section.

In Section 3, the test setup of the UWB system and the machine tool was explained and visualized. In a total of four scenarios, the signal propagation was improved by gradually increasing the active lines of sight between the tag and the anchors, reducing the influence of other interfering objects on the indoor positioning system. A high-precision Faro Vantage S6 laser tracker was used as a reference measurement device.

The data evaluation of the recorded UWB data in reference to the laser tracker data in Section 4 was able to prove a clear influence of the active lines of sight connections and the presence of the machine tool on the accuracy of the indoor positioning system. The average deviation of the UWB technology could be improved by a factor of 14 from scenario 1 (two lines of sight) to scenario 4 (four lines of sight in a free environment) from 87.64 cm ± 32.27 cm to 6.07 cm ± 2.24 cm (confidence interval: 95%). Consequently, in any use case, the positioning and density of the anchor modules in relation to the number of line of sight connections to the tag and potential interference contours should be the highest priority in the design of a UWB system. The higher the number of line of sight connections between the anchors and the tag, and the fewer interfering objects in and around the measurement range of the indoor positioning system, the higher its accuracy in determining the position becomes.

## 6. Conclusions and Further Research

In the research work of this article, the measurement uncertainty and the confidence intervals for the used indoor positioning systems in the environment of a metal machine tool were determined. To analyse whether a measurement device is suited for a specific application, the device must be evaluated in the context of its use. An estimate to judge about the suitability of a measurement device is known as the golden rule of metrology, which claims that the measurement uncertainty needs to be smaller than 1/10 of the required tolerance of a feature [31]. In the related work of the authors of this article, the indoor positioning system was used to track the position of a worker to find out if a machine is in changeover. The shoulder breadth as a tolerance width for tracking a male person of an age between 18 and 65 years within the 95% percentile is 52.5 cm [32]. Accordingly, the measurement uncertainty of a suited measurement device should be less than 5.25 cm. Referring to Table 3, only scenario 3 is suited for the mentioned tracking application with a measurement uncertainty of 2.16 cm. Scenarios 1 and 2 offer a higher measurement uncertainty of 15.74 cm (scenario 1) and 7.24 cm (scenario 2), respectively. Scenario 4 is the ideal scenario without the machine tool as a signal obstacle. With reference to Figure 7, this implies that for the tracking of workers in the area of the metal machine tool four lines of sight are required for the positioning system. In conclusion, the selected UWB system is very well suited for industrial use cases with high precision positioning requirements, if the number of lines of sight and the influence of interfering objects are considered in the design of the use case.

To explore the reason for the higher y-deviation in relation to the x-deviation described in Section 4.5, there is a need for further investigation. In addition, evaluation of the influence of the anchor heights, was not investigated in detail in this experiment. A further investigation approach is also the variation of the measurement method of the UWB system from two-Way ranging to the time difference of arrival (TDoA) method, which is also frequently used.

## Figures and Tables

**Table 1:** Overview of radio frequency technologies for indoor positioning [3].

Technology	Accuracy [m]	Range [m]	Frequency [GHz]
WLAN/Bluetooth	0.9–10	1–100	2.4 [4]
Ultra-wideband (UWB)	0.1–1	1–90	3.1–10.6 [4]
Ultrasound	0.01–0.09	1–20	>$20\cdot 10^{-6}$ [5]

**Table 2:** Impact factors on indoor positioning systems (factors indicated by * are fixed in the experiment setup).

Signal Propagation	Signal Transmission	Signal Reception	Signal Processing
Building structure *	Anchor density	Operators *	Filter *
Building material *	Anchor distribution	Receivers/Tag *	
Furniture placement	Anchor heights *	Sampling *	
People crowed *	Anchor models *	Number of samples *	
Temperature *			
Humidity *			

**Table 3:** Comparison of the scenarios.

		Scenario 1	Scenario 2	Scenario 3	Scenario 4
Mean value μ [cm]	x	75.02	10.99	5.37	2.70
	y	91.11	55.89	15.90	13.83
	total	141.18	58.96	17.78	14.42
Standard deviation σ [cm]	x	109.66	11.97	3.97	2.58
	y	43.12	41.48	12.82	6.33
	total	87.64	40.30	12.02	6.07
Standard uncertainty $u_{A}$ [cm]		15.74	7.24	2.16	1.09
Confidence interval [cm]		87.64 ± 32.27	40.30 ± 14.84	12.02 ± 4.43	6.07 ± 2.24
(Confidence level γ=95%)					

## Data Availability

The underlying datasets are available under https://github.com/neubertill/data_indoor-GPS.git (accessed on 14 December 2022).

## Abbreviations

The following abbreviations are used in this manuscript:

BLE	Bluetooth Low Energy
CSV	Comma Separated Values
GNSS	Global navigation satellite system
GUM	Guide to the expression of uncertainty in measurement
LOS	Line-of-Sight
NLOS	Non-Line-of-Sight
RSS	Received Signal Strength
TWR	Two-Way Ranging
UWB	Ultra-Wideband

## Appendix A

The technical specifications of the used hardware is detailed in Table A1.

**Table A1:** Technical information.

Device	Manufacturer	Device Name
Milling machine	Spinner	U5-620
Machine control	Siemens	840D-SL V4.4
Transmitter	Localino	Localino Anchor v4.0
Receiver	Localino	Localino v3.0 Tag
Laser rangefinder	Bosch	GLM 100-25 C PROFESSIONAL
Laser tracker	Faro	Vantage S6
